# Differential Improvement in Obsessive Rumination and Fantasy Intrusions Versus Attachment-Related Emotional Dysregulation During Oral Glutamatergic Augmentation in a 16-Year-Old Adolescent

**DOI:** 10.7759/cureus.112492

**Published:** 2026-07-11

**Authors:** Hoi Ki Cheung, Ngo Cheung

**Affiliations:** 1 Department of Psychiatry, Chinese University of Hong Kong, Hong Kong, HKG; 2 Department of Psychiatry, Cheung Ngo Medical Limited, Hong Kong, HKG

**Keywords:** adolescent obsessive-compulsive symptoms, cyp2d6 inhibition, dextromethorphan, glutamatergic augmentation, piracetam

## Abstract

A 16-year-old post-pubertal female presented with mixed anxiety-depressive symptoms, prominent obsessive rumination, relationship-centered guilt, reassurance-seeking, fantasy intrusions, and difficulty separating fantasy from reality during periods of distress. Baseline scores were in the moderate range for depression and severe range for anxiety, with a Patient Health Questionnaire-9 score of 14 and a Generalized Anxiety Disorder-7 score of 20. A low-to-moderate-intensity Cheung glutamatergic regimen-like strategy was introduced, consisting of dextromethorphan, a CYP2D6-inhibiting antidepressant, and piracetam, with risperidone co-prescribed for fantasy-related cognitions, overvalued ideas, and obsessive instability. Across the follow-up period from January to June 2026, depressive symptoms improved to the minimal-to-mild range, and anxiety symptoms improved to the mild range, with Patient Health Questionnaire-9 scores mostly between 4 and 6 and Generalized Anxiety Disorder-7 scores mostly between 6 and 7, except for one stress-related rise to 11. Obsessional symptoms reportedly reduced by more than 30% after dextromethorphan escalation and piracetam introduction. Later improvements included better ability to interrupt rumination, reduced preoccupation with “pretending to be ill,” shorter fantasy intrusions, and improved fantasy-reality differentiation. No medication side effects, dissociation, hypomania, serotonin-toxicity symptoms, sleep disruption, appetite loss, or renewed self-harm ideation were documented. The clinically distinctive feature was a discordant response pattern as follows: repetitive, obsessive, and fantasy-related intrusions appeared to loosen more clearly than attachment-linked emotional reactivity. The course is compatible with the hypothesis that N-methyl-D-aspartate and α-amino-3-hydroxy-5-methyl-4-isoxazole propionic acid (NMDA-AMPA) modulation may help selected post-pubertal adolescents disengage from rigid perseverative loops. However, interpretation is limited by polypharmacy, risperidone titration, selective serotonin reuptake inhibitor (SSRI) effects, supportive care, natural symptom fluctuation, and the absence of formal obsessive-compulsive ratings or withdrawal/rechallenge. This case is best viewed as hypothesis-generating.

## Introduction

Adolescents with mixed anxiety-depressive presentations often do not present with mood and worry alone. In routine practice, the most impairing symptoms may be repetitive guilt, intrusive doubts, confession urges, reassurance-seeking, relationship-centered emotional storms, and fears about the meaning of one’s own thoughts. Some patients also describe fantasy-proneness, intense daydreaming, or brief difficulty separating imagined scenarios from real events when distressed. These features may overlap with obsessive-compulsive spectrum symptoms even when the initial working diagnosis is mixed anxiety-depressive disorder.

First-line care for adolescent anxiety, depression, and obsessive-compulsive disorder usually involves psychoeducation, family involvement, cognitive-behavioral therapy, exposure and response prevention when obsessive-compulsive disorder (OCD) is present, and serotonin reuptake inhibitors when medication is indicated. These approaches remain the standard of care [1,2]. Still, a subset of patients has persistent “stuck-loop” symptoms as follows: repetitive doubt, mental checking, guilt-based rumination, and repeated attempts to obtain certainty. The glutamatergic system has therefore attracted interest in OCD and related perseverative states, particularly because abnormalities in glutamate signaling have been implicated in obsessive-compulsive disorder and because N-methyl-D-aspartate (NMDA)-modulating agents have shown anti-obsessional effects in early studies [3-5].

A related but more concise rationale comes from rapid-acting antidepressant research. Ketamine-class work suggests that rapid behavioral effects may involve α-amino-3-hydroxy-5-methyl-4-isoxazole propionic acid (AMPA)-dependent downstream plasticity rather than NMDA blockade alone [6-9]. These findings have encouraged attempts to separate the plasticity-related signal from the dissociative and logistical burdens of ketamine.

The Cheung glutamatergic regimen (CGR) has been proposed as an oral, ketamine-class strategy that uses dextromethorphan for NMDA modulation, CYP2D6 inhibition to prolong dextromethorphan exposure, piracetam to facilitate AMPA receptor throughput, and glutamine as a substrate in the full four-component model [10]. The present case did not use the full four-component regimen because glutamine was not included. It used a CGR-like core of dextromethorphan, CYP2D6-inhibiting antidepressant exposure, and piracetam, with risperidone added for obsessive and fantasy-related cognitions. Piracetam has been discussed as a practical AMPA-facilitating component because of its long clinical history and proposed effects on AMPA receptor function, although its human psychiatric evidence remains limited [11-13].

Developmental timing is important. Adolescence is still a period of synaptic refinement, emotional maturation, and vulnerability to activation, but it differs from early childhood. General neurodevelopmental literature describes adolescence as a period of ongoing pruning and circuit reorganization [14,15]. CGR-related developmental hypotheses argue that glutamatergic enhancement should be avoided in young children with active synaptic surplus, especially in autism-related contexts, but may be more plausible after puberty when carefully titrated and closely monitored [16-18]. This case is presented to describe, not prove, the clinical course of a post-pubertal adolescent treated with a CGR-like approach in a real-world setting. The specific knowledge gap addressed here is the clinical description of a CGR-like oral approach in a post-pubertal adolescent whose most impairing symptoms were obsessive and fantasy-related perseverative loops, not depression or anxiety scores alone.

## Case presentation

The patient was a 16-year-old Form 5 female student who self-referred through an online platform and attended the first consultation with her elder sister (Table 1). She reported no major academic pressure and no difficulty with formal presentations. Appetite was generally preserved. Her background included multiple somatic complaints, significant social anxiety, and marked self-consciousness about appearance.

**Table 1:** Clinical course of a 16-year-old adolescent female treated with a CGR-like glutamatergic augmentation strategy. This table is descriptive and hypothesis-generating only. The clinical course involved concurrent SSRI, DXM, piracetam, and risperidone titration; therefore, improvement cannot be attributed to any single component. CGR: Cheung glutamatergic regimen; DXM: dextromethorphan; GAD-7: Generalized Anxiety Disorder-7; PHQ-9: Patient Health Questionnaire-9; SSRI: selective serotonin reuptake inhibitor; CR: controlled release

Day/phase	Clinical context	Dominant symptoms and interval change	Rating scales	Medication regimen/change	Course interpretation
Day 147 to day 56, pre-treatment	Breakup after approximately one-year relationship; later disclosure of multiple lies during relationship; resumed communication due to guilt.	Emotional instability, repeated texting/scolding of ex-boyfriend, intrusive guilt, worry about future consequences, self-harm in October, persistent relationship-centered dysregulation.	Not recorded	None documented.	Interpersonal trigger preceded onset of obsessive guilt, affective instability, and self-harm behavior.
Day 0, consultation 1	Initial presentation with elder sister. Mixed anxiety-depressive disorder diagnosed; OCD to monitor/rule out.	Frequent intrusive thoughts, uncontrollable rumination, habitual fantasy/daydreaming, marked pre-sleep anxiety, social anxiety, image-consciousness, low mood and motivation. Vague fantasy-reality boundary noted.	PHQ-9: 14; GAD-7: 20	Alprazolam 0.25 mg daily × 5 days; fluoxetine 10 mg morning; dextromethorphan 30 mg at night.	Severe anxiety and moderate depression at baseline; CGR-like NMDA component initiated with CYP2D6-inhibiting SSRI.
Day 3, consultation 2	Encountered ex-boyfriend at school; scolded him and cried.	Persistent self-blame, intrusive thoughts unchanged, constant questioning of words/actions, distress about appearing odd. Motivation and appetite showed early improvement. No self-harm urges, but hitting self during anger disclosed.	Not recorded	Fluoxetine increased to 20 mg in the morning; risperidone 0.25 mg at night added; dextromethorphan 30 mg at night continued.	Early tolerability with partial motivational improvement; antipsychotic augmentation added for rumination/fantasy-related instability.
Day 15, consultation 3	Ongoing relationship-centered rumination and reassurance/confession urges.	Mood improved and self-hitting urges reduced, but intrusive thoughts persisted as follows: whom ex-boyfriend met, whether she loved others, need to tell her ex-boyfriend everything, fear of faking illness. Sexual/fantasy cognitions became distressing and were resisted.	PHQ-9: 9; GAD-7: 16	Fluoxetine 20 mg; risperidone 0.25 mg; dextromethorphan increased to 45 mg at night; piracetam 300 mg at night added.	Depressive symptoms improved; obsessive/fantasy symptoms persisted. AMPA-facilitating component introduced.
Day 29, family visit	Mother attended; medication rationale and monitoring were explained.	Obsessional symptoms reduced by more than 30%. Urges to hit self less frequent. Persistent rumination about past actions and pretending/faking illness. One intense nocturnal self-blame episode with repeated calls to ex-boyfriend. Mild morning fatigue.	Not recorded	Fluoxetine 20 mg; risperidone 0.25 mg; dextromethorphan approximately 60 mg at night; piracetam split into 300 mg in the morning and 300 mg at night.	First clear reduction in obsessional burden after DXM escalation and piracetam addition, though residual interpersonal dysregulation remained.
Day 50, consultation 5	Mood strongly linked to ex-boyfriend’s mood and contact.	Persistent obsession with “pretending to be ill.” Thoughts that others had fallen in love with her caused distress. Continued episodic scolding of ex-boyfriend. Sleep and appetite preserved.	Not recorded	Fluoxetine 20 mg; risperidone increased to 1 mg at night; dextromethorphan 15 mg in the morning and 45 mg at night; piracetam 300 mg in the morning and 300 mg at night.	Persistent obsessive/fantasy-related cognitions prompted risperidone increase and split DXM dosing.
Day 64, consultation 6	Post-adjustment review after risperidone increases and split DXM regimen.	More able to stop obsessive rumination. No longer preoccupied with acting/pretending to be ill. Mood more stable even without ex-boyfriend contact. Better differentiation of fantasy from reality. Residual ego-syntonic ruminations persisted.	PHQ-9: 4; GAD-7: 7	Fluoxetine moved to night dosing; risperidone 1 mg at night; dextromethorphan increased to 30 mg in the morning and 45 mg at night; piracetam 300 mg in the morning and 300 mg at night.	Major improvement in depression, anxiety, rumination control, and fantasy-reality discrimination. No side effects reported.
Day 78, consultation 7	Functioning more stable, though contact with ex-boyfriend continued.	Mood improved; less preoccupied with “main character syndrome.” Fantasy thoughts about other males were reduced from repetitive/frequent to only a few seconds. Mild, manageable difficulty waking. Ongoing attachment to ex-boyfriend with insight into low likelihood of reconciliation.	PHQ-9: 5; GAD-7: 7	Continued fluoxetine 20 mg at night; risperidone 1 mg at night; dextromethorphan 30 mg in the morning + 45 mg at night; piracetam 300 mg in the morning + 300 mg at night.	Sustained symptomatic improvement; residual relational dependency persisted despite reduced obsessive/fantasy intrusions.
Day 106, consultation 8	Interpersonal stress at school: confidential school communication issued.	Easily irritated by ex-boyfriend. One public emotional dysregulation episode after crying and demanding ex-boyfriend’s presence. Evening low mood is prominent. Rumination about other males has reduced. Sleep and appetite preserved.	PHQ-9: 6; GAD-7: 11	No prescription changes documented.	Interpersonal trigger caused partial anxiety worsening, but overall functioning remained improved relative to baseline.
Day 113, prescription visit	Ex-boyfriend refused reconciliation; patient began daily communication with another male.	Developing emotional attachment to new male. Some insight into emotional dependency. Deliberately spent time with new male in front of ex-boyfriend. Residual fantasy cognitions toward new male. Mood more stable; sleep adequate.	PHQ-9: 4; GAD-7: 6	Fluoxetine 20 mg at night; risperidone increased to 1.5 mg at night; dextromethorphan 45 mg at night; piracetam 300 mg in the morning + 300 mg at night.	Mood and anxiety remained minimal/mild despite relationship transition; risperidone was increased for residual fantasy/attachment-related cognitions.
Day 141, consultation 10	Examination period; possible plan to reconcile after exams.	Mood generally stable; cried over trivial matters. Sleep and appetite are okay. New symptom: associative rumination triggered by wording or phrases. No medication side effects reported.	PHQ-9: 5; GAD-7: 6	Fluoxetine 20 mg at night; risperidone 1.5 mg at night; dextromethorphan 45 mg at night; piracetam 300 mg morning + 300 mg at night; paroxetine CR 3.125 mg at night added; loratadine 10 mg daily.	Sustained mild-range symptoms with residual associative rumination; added ultra-low-dose paroxetine increased CYP2D6-inhibiting/serotonergic load.

The main interpersonal trigger began in September 2025, when she ended an approximately one-year relationship because she felt she did not truly “love” her boyfriend. Although she had initiated the breakup, she became frustrated when she saw him appearing happy and repeatedly scolded him. In October 2025, the ex-boyfriend discovered that she had told many lies during the relationship, including about past relationships and other personal matters. Her emotional state became unstable. She repeatedly texted and scolded him, developed intrusive worries that her past actions might affect her future, worried that she was wasting time, and engaged in self-harm. In November 2025, communication resumed because she felt guilty. The pattern of guilt, repeated contact, and emotional instability continued.

At the first consultation on day 0, she described frequent intrusive thoughts that were sometimes difficult to control, habitual fantasy and daydreaming, anxiety about the future, marked pre-sleep anxiety, low mood, and low motivation. Sleep onset was not prolonged, and there was no midnight awakening. She was socially anxious and highly concerned about her image. Relationship-centered distress was prominent. The ex-boyfriend had texted that he would find another girl. When upset, she would speak with her elder sister, but afterward she would worry that she had fallen in love with her sister. She imagined alternative endings to dramas and had many fantasies. A vague boundary between fantasy and reality was noted.

Her baseline Patient Health Questionnaire-9 score was 14, in the moderate range, with prominent guilt/self-critical thoughts and concentration difficulty; the self-harm item was scored as zero. Her Generalized Anxiety Disorder-7 score was 20, in the severe range, with near-daily nervousness, uncontrollable worry, excessive worry, difficulty relaxing, restlessness, and irritability. The initial clinical impression was mixed anxiety-depressive disorder, with obsessive-compulsive disorder to be monitored or ruled out. She was prescribed alprazolam 0.25 mg daily for five days, fluoxetine 10 mg in the morning for seven days, and dextromethorphan 30 mg at night.

At the second consultation on day 3, medication was tolerated. She had encountered her ex-boyfriend at school, scolded him, and cried. Self-blame persisted. Intrusive thoughts were unchanged in frequency. She constantly questioned her words and actions and was distressed by the possibility of appearing odd to others. Motivation and appetite showed early improvement. She denied self-harm urges but disclosed hitting herself during anger. Fluoxetine was increased to 20 mg in the morning. Risperidone 0.25 mg at night was added. Dextromethorphan 30 mg at night was continued.

At the third consultation on day 15, she was purchasing melatonin on her own for sleep. Urges to hit herself had reduced, and mood had improved, but intrusive and worrisome thoughts persisted. She reported needing more attention from the boyfriend or ex-boyfriend and feeling compelled to tell him everything. Intrusions included thoughts about whom the ex-boyfriend had met, whom she herself had met, whether she had fallen in love with someone else, and whether she had faked her illness. “Main character syndrome” was queried. She described sexual thoughts toward males since Form 1 and sexual fantasies toward another male even during the relationship. These fantasies were easily triggered by voices, including at home. Initially she enjoyed the image, but later she found it distressing, tried to stop it, and wanted clarification about whether she had intentionally thought it. Her Patient Health Questionnaire-9 score improved to 9 and her Generalized Anxiety Disorder-7 score to 16. Dextromethorphan was increased to 45 mg at night, and piracetam 300 mg at night was added, while fluoxetine 20 mg and risperidone 0.25 mg were continued.

On day 29, the patient’s mother contacted the clinic to ask why the medications were being increased, what the intended effects were, what side effects might occur, and how long the treatment would continue. She was invited to attend in person, and the illness and monitoring plan were discussed. At this visit, obsessional symptoms had reduced by more than 30%. Urges to hit herself were less frequent. She still ruminated about past actions and continued to question whether she had fabricated her illness. One intense nocturnal episode involved extensive rumination about having lied to the ex-boyfriend and repeated calls to him throughout the night. Mild morning fatigue was reported. Fluoxetine 20 mg and risperidone 0.25 mg were continued. Dextromethorphan was prescribed at approximately 60 mg nightly, and piracetam was split to 300 mg in the morning and 300 mg at night.

At the day 50 visit, she remained highly preoccupied with whether she was “pretending to be ill.” She often thought that others had fallen in love with her, which caused distress. Talking with her ex-boyfriend made her feel better, but she still became emotionally unstable and scolded him at times. Sleep and appetite were preserved, and mood remained strongly tied to the ex-boyfriend’s mood and attitude. Risperidone was increased to 1 mg nightly to target obsessive-compulsive and fantasy-related cognitions. Dextromethorphan was split to 15 mg in the morning and 45 mg at night. Piracetam was maintained at 300 mg twice daily, and fluoxetine 20 mg was continued.

The most marked clinical change was documented on day 64. She was more able to stop obsessive rumination and no longer thought as much about acting or pretending to be ill. Overall control improved. She still called and scolded the ex-boyfriend about weekly when she felt out of control, unhappy, or sad about him, but mood was more stable even without contact. She was better able to differentiate fantasy from reality. Residual ruminations persisted and were described as ego-syntonic. Her Patient Health Questionnaire-9 score fell to 4 and her Generalized Anxiety Disorder-7 score to 7. No side effects were reported. Fluoxetine was moved to nighttime dosing. Dextromethorphan was increased to 30 mg in the morning and 45 mg at night. Risperidone 1 mg nightly and piracetam 300 mg twice daily were continued.

At the day 78 visit, mood remained improved. She was less preoccupied with “main character syndrome.” Sleep was okay, with mild but manageable difficulty waking. She still regularly sought out or contacted the ex-boyfriend and believed she still liked him, while recognizing that reconciliation was unlikely because of trust issues. She continued to notice other males and had fantasy thoughts, but these had reduced from repetitive and frequent episodes to only a few seconds at a time. Functioning was more stable. Her Patient Health Questionnaire-9 score was 5, and Generalized Anxiety Disorder-7 score was 7. No side effects were reported, and the regimen was continued.

On day 106, interpersonal stress became prominent again. She was easily irritated by the ex-boyfriend. One public episode occurred in which she displayed her temper, cried, and demanded his immediate presence. Evening low mood was more noticeable. Sleep onset and appetite remained preserved. Rumination about other males had reduced. A confidential communication was issued to the school, stating that mood and daily functioning had stabilized compared with baseline; interpersonal sensitivity remained a vulnerability; intermittent mood fluctuations and rumination were manageable; she had good self-awareness; she was willing to confide in family; and no immediate safety concerns were identified. The school was encouraged to provide space and understanding during interpersonal stress. Her Patient Health Questionnaire-9 score was 6, and her Generalized Anxiety Disorder-7 score rose to 11. No prescription change was documented.

On day 113, the ex-boyfriend formally indicated that he would not reconcile. The patient began daily communication with another male and appeared to be developing emotional attachment. Parents were informed at a school parents’ day event. She showed some insight into her emotional dependency, though she was noted to spend time with the new male in front of the ex-boyfriend. Residual fantasy cognitions toward the new male were present. Sleep was adequate, and mood was more stable. Her Patient Health Questionnaire-9 score was 4, and her Generalized Anxiety Disorder-7 score was 6. Risperidone was increased to 1.5 mg nightly. Dextromethorphan was reduced to 45 mg nightly, while fluoxetine 20 mg nightly and piracetam 300 mg twice daily were continued.

At the last documented visit on day 141, during the examination period, mood was generally stable, although she cried over trivial matters. Sleep and appetite were okay. She was considering getting back together with her boyfriend after examinations. A new symptom was identified as follows: associative rumination triggered by wording or phrases. For example, hearing a single phrase could lead her to ruminate on a related or distressing idea. Her Patient Health Questionnaire-9 score was 5, and her Generalized Anxiety Disorder-7 score was 6. The self-harm item remained zero. No medication side effects were reported. The regimen consisted of fluoxetine 20 mg nightly, risperidone 1.5 mg nightly, dextromethorphan 45 mg nightly, piracetam 300 mg morning and night, and newly added paroxetine controlled-release 3.125 mg nightly. Loratadine 10 mg daily was also prescribed for 10 days.

## Discussion

**Figure 1:**
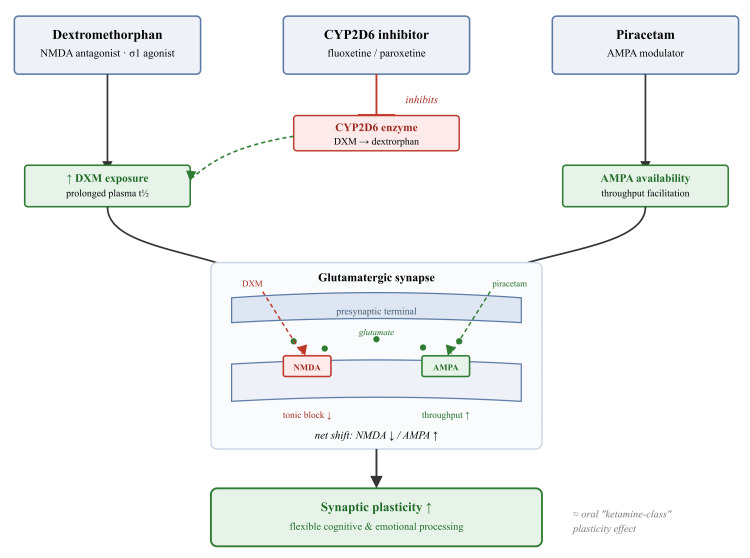
Proposed pharmacological mechanism of the Cheung glutamatergic regimen (CGR). This conceptual figure is hypothesis-generating and should not be read as evidence that the regimen produces clinical benefit. Three oral agents act in concert. Dextromethorphan (DXM) provides NMDA-receptor antagonism with σ1-receptor agonism; a CYP2D6-inhibiting antidepressant (fluoxetine or paroxetine) blocks CYP2D6-mediated conversion of DXM to dextrorphan, prolonging plasma exposure to DXM and its active metabolites; and piracetam facilitates AMPA-receptor throughput. At the glutamatergic synapse, this produces a coordinated shift in the NMDA-AMPA balance, with a relative reduction in tonic NMDA signaling alongside enhanced AMPA-mediated transmission, which is hypothesized to upregulate synaptic plasticity and support more flexible cognitive and emotional processing, conceptually paralleling the rapid plasticity effects of ketamine-class agents via an accessible oral route. The scheme is mechanistic and hypothesis-generating; it does not depict clinical outcomes. This image was created by the author (Ngo Cheung) of this study using PowerPoint (Redmond, WA: Microsoft Corp.), and no AI was used. AMPA: α-amino-3-hydroxy-5-methyl-4-isoxazole propionic acid; NMDA: N-methyl-D-aspartate

This case describes a post-pubertal adolescent with mixed anxiety-depressive symptoms, prominent obsessive rumination, fantasy intrusions, and relationship-driven dysregulation who improved during treatment with a Cheung glutamatergic regimen (CGR)-like glutamatergic augmentation strategy combined with fluoxetine and risperidone (Figure 1). The improvement was clinically meaningful. Quantitatively, the PHQ-9 decreased from 14 at day 0 to 4 at day 64 and then remained between 4 and 6 through day 141, while the GAD-7 decreased from 20 at day 0 to 7 at day 64 and then stayed between 6 and 7 except for a stress-related rise to 11 at day 106. Depression scores moved from the moderate range to the minimal or mild range, and anxiety scores moved from severe to the mostly mild range. Self-harm urges and self-hitting urges reduced. Obsessional symptoms reportedly fell by more than 30% after dextromethorphan escalation and piracetam introduction. Later, she became more able to interrupt rumination, stopped focusing so intensely on whether she was “pretending to be ill,” and showed improved fantasy-reality differentiation.

**Figure 2:**
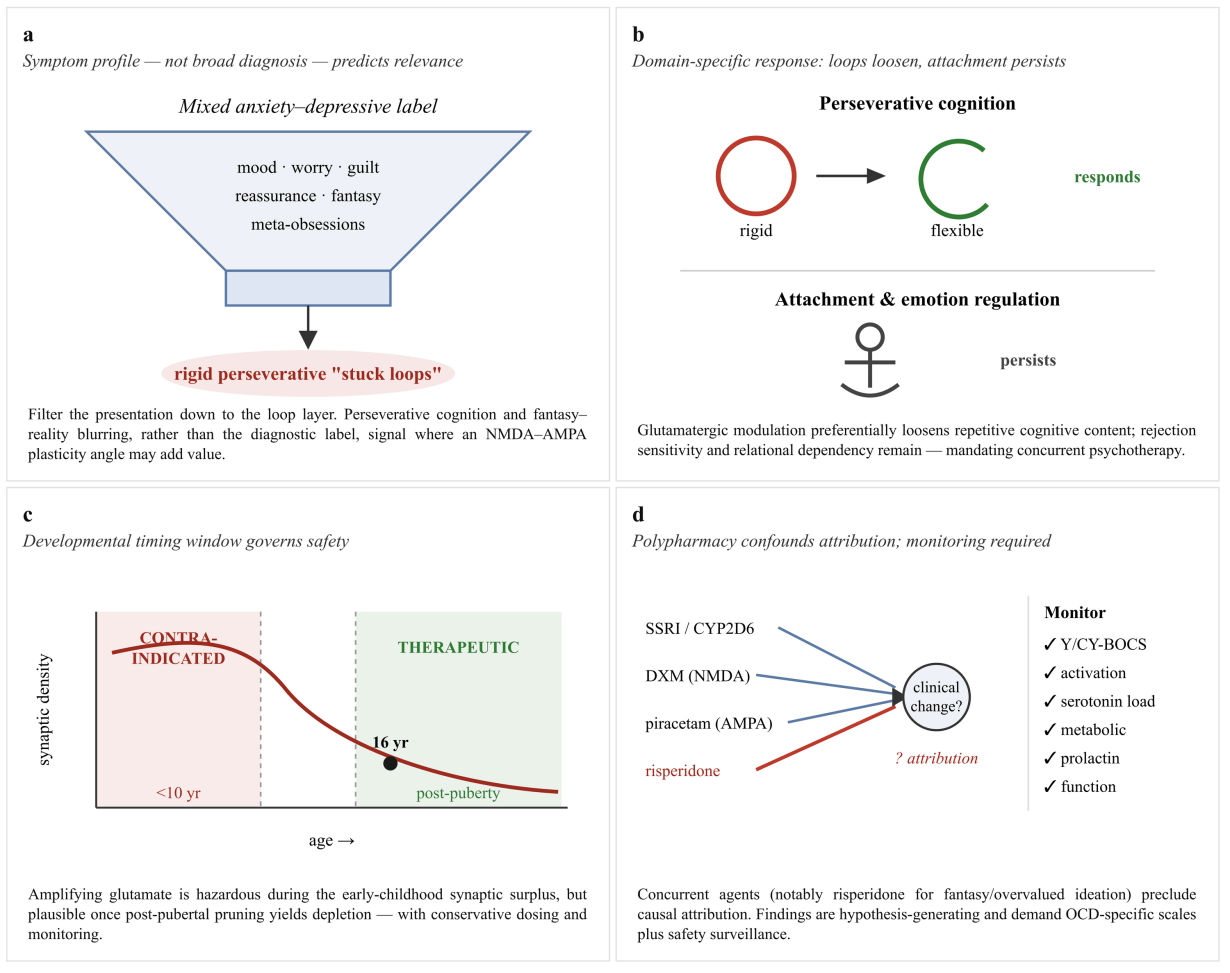
Conceptual teaching principles for CGR-like oral glutamatergic augmentation (dextromethorphan + CYP2D6-inhibiting antidepressant + piracetam, ± low-dose antipsychotic) in a post-pubertal adolescent with perseverative and fantasy-related symptoms. This conceptual teaching figure is not a validated treatment algorithm. (a) The symptom profile, particularly rigid perseverative loops and fantasy-reality blurring, rather than the broad diagnostic label, identifies candidates for a glutamatergic plasticity strategy. (b) Responses are domain-specific - repetitive cognitive content tends to loosen while attachment-related emotional dysregulation persists. (c) Developmental timing is decisive - the regimen is contraindicated during the early-childhood synaptic surplus but theoretically plausible after pubertal pruning. (d) Multi-agent regimens prevent causal attribution and require structured, OCD-specific and metabolic monitoring; such observations remain hypothesis-generating. This image was created by the author (Ngo Cheung) of this study using PowerPoint (Redmond, WA: Microsoft Corp.), and no AI was used. OCD: obsessive-compulsive disorder; NMDA: N-methyl-D-aspartate; AMPA: α-amino-3-hydroxy-5-methyl-4-isoxazole propionic acid; SSRI: selective serotonin reuptake inhibitor; DXM: dextromethorphan

The pattern is compatible with, but does not prove, the CGR hypothesis (Figures 2a-2d). Cheung proposed that dextromethorphan, when prolonged through CYP2D6 inhibition, may provide an oral NMDA-modulating “spark,” while piracetam may facilitate AMPA-mediated throughput [10]. This model is grounded in ketamine-related evidence suggesting that rapid clinical effects are not simply due to NMDA blockade but also depend on AMPA activation and downstream plasticity [7-9]. In the present case, the most relevant symptoms were not only low mood and worry, but repetitive cognitive loops - guilt, confession urges, illness-doubt, questions about whether thoughts were intentional, fantasy intrusions, and word-triggered associative rumination. These symptoms fit the “stuck-loop” formulation better than a broad depression-plus-anxiety formulation alone.

The response was domain-specific. Perseverative and obsessional symptoms improved most clearly, including intrusive thoughts, self-blame loops, illness-doubt rumination, fantasy intrusions, and general anxiety/mood symptoms. In contrast, attachment-related dysregulation was more persistent. She continued to seek contact with the ex-boyfriend, became distressed by perceived rejection, scolded him during emotional episodes, and later shifted attachment toward another male. One public dysregulation episode occurred in May despite otherwise improved scores. This distinction is clinically useful. A glutamatergic strategy, if active, may have helped reduce repetitive cognitive “stickiness,” while relational dependency, rejection sensitivity, and emotion regulation required ongoing psychosocial work.

The timing of improvement also deserves attention. Early treatment with fluoxetine and low-dose dextromethorphan was associated with partial improvement in mood, motivation, appetite, and self-harm urges. More obvious anti-obsessional improvement occurred after dextromethorphan was increased and piracetam was added. A larger turning point followed risperidone escalation to 1 mg nightly, split dextromethorphan dosing, and continued split piracetam dosing. By March 31, the patient’s Patient Health Questionnaire-9 score had fallen to 4 and Generalized Anxiety Disorder-7 score to 7, and she was better able to stop rumination and distinguish fantasy from reality. This temporal clustering is consistent with CGR case-series themes around titration and split dosing [19,20]. However, it remains only a temporal association.

Risperidone is a major confounder and should not be minimized. The most dramatic improvement in fantasy-reality differentiation and reduction of overvalued ideas occurred after risperidone was increased from 0.25 mg to 1 mg. Risperidone has evidence as an augmentation strategy in treatment-resistant OCD and related presentations, especially when intrusive or overvalued content is prominent [21-23]. In this case, any claim that dextromethorphan or piracetam alone improved fantasy-related symptoms would be unjustified. The most balanced interpretation is that the CGR-like components may have contributed to reduced perseveration, while risperidone likely contributed substantially to stabilization of fantasy-proneness, overvalued ideation, and blurred boundaries.

The patient’s age is also relevant. At 16 years, she falls into a post-pubertal developmental window in which CGR timing hypotheses consider carefully titrated glutamatergic enhancement more plausible than in young children [16,17]. This does not mean the approach is risk-free. Adolescence remains a period of ongoing synaptic remodeling, emotional reactivity, and vulnerability to activation [14,15]. Consent, parental involvement, careful titration, and close monitoring are especially important. In this case, the mother’s questions about medication burden, intended effects, side effects, and treatment duration were clinically appropriate, and the decision to discuss these in person was a useful part of risk management.

Safety issues deserve emphasis. Dextromethorphan has serotonergic properties, and fluoxetine and paroxetine can inhibit CYP2D6, increasing dextromethorphan exposure. The subsequent addition of ultra-low-dose controlled-release paroxetine on top of fluoxetine and dextromethorphan was pharmacologically meaningful, even at a small dose. CYP2D6 inhibition can be useful when deliberately prolonging dextromethorphan exposure, but it also increases the risk of drug interactions and serotonin toxicity [24-27]. In this case, no serotonin-toxicity symptoms, dissociation, hypomania, appetite loss, sleep deterioration, or subjective medication side effects were documented. This is reassuring but not definitive.

Risperidone adds a different monitoring burden. In adolescents, second-generation antipsychotics require attention to weight, body mass index, blood pressure, glucose or HbA1c, lipids, extrapyramidal symptoms, menstrual changes, and prolactin-related effects where clinically indicated [28,29]. The provided record did not document metabolic or prolactin measurements. Future similar cases should include these measures from baseline onward, especially when risperidone is increased to 1 mg or above and maintained.

The rating scales used here were helpful but incomplete. The Patient Health Questionnaire-9 and Generalized Anxiety Disorder-7 are practical tools for tracking depressive and anxiety symptoms [30,31]. In this patient, they captured a large improvement in mood and anxiety. They did not fully capture the most distinctive symptoms: intrusive guilt, confession urges, reassurance-seeking, fantasy intrusions, and associative rumination. The reported greater-than-30% reduction in obsessional burden was therefore a clinical estimate rather than a score derived from the Children’s Yale-Brown Obsessive Compulsive Scale. A future case series should include the Children’s Yale-Brown Obsessive Compulsive Scale or Yale-Brown Obsessive Compulsive Scale, depending on age and setting, along with activation screening, family collateral, school functioning, peer functioning, and specific tracking of fantasy-reality differentiation [32,33].

The residual symptoms are also informative. Despite overall improvement, interpersonal dependency persisted. Rejection by the ex-boyfriend remained destabilizing, and attachment appeared to shift toward another male. By June, a new symptom appeared: word- or phrase-triggered associative rumination. This may represent an incomplete anti-obsessional response, a separate semantic-rumination subtype, or a cognitive style that requires targeted psychotherapy. Exposure and response prevention, cognitive therapy focused on intolerance of uncertainty, and family-supported reduction of reassurance cycles would be reasonable non-pharmacological priorities [1,34]. Adjunctive approaches such as N-acetylcysteine have been studied in refractory OCD and related disorders, but any use in this context would remain speculative and would require careful safety assessment [35,36].

This case has major limitations. It was naturalistic and uncontrolled. Several active agents were used as follows: fluoxetine, later ultra-low-dose paroxetine controlled-release, dextromethorphan, piracetam, risperidone, brief alprazolam, and melatonin purchased by the patient. Medication changes overlapped with changes in relationship circumstances, family involvement, school support, and the natural course of adolescent distress. There was no placebo control, no formal obsessive-compulsive scale, no structured diagnostic interview, no withdrawal/rechallenge, and no blinded assessment. The apparent improvement cannot be attributed to any single component. The most defensible conclusion is that the clinical trajectory is consistent with CGR-related hypotheses and supports prospective study, not that the regimen is proven effective.

## Conclusions

This longitudinal case describes clinically meaningful improvement in a 16-year-old adolescent with mixed anxiety-depressive disorder, prominent obsessive-compulsive spectrum rumination, fantasy intrusions, and attachment-related dysregulation during treatment with a low-to-moderate-intensity CGR-like regimen. Depression and anxiety scores moved from moderate and severe baseline ranges to mostly minimal or mild ranges. Obsessional symptoms, illness-doubt rumination, self-harm urges, and intrusive fantasy content improved, and fantasy-reality differentiation became clearer. The case is useful because the response was not global. Perseverative cognitive loops improved more than attachment-related emotional dysregulation. This suggests that symptom profile may matter more than broad diagnosis when considering glutamatergic hypotheses. At the same time, risperidone was a major confounder, especially for fantasy-related and overvalued cognitions, and the SSRI contribution cannot be separated from the dextromethorphan and piracetam components.

The report should therefore be read as hypothesis-generating. It supports careful prospective study of oral glutamatergic augmentation in selected post-pubertal adolescents, with conservative dosing, family involvement, structured monitoring, OCD-specific scales, metabolic and prolactin surveillance when antipsychotics are used, and psychotherapy targeting relationship patterns and reassurance cycles.
